# TG01/GM-CSF and adjuvant gemcitabine in patients with resected RAS-mutant adenocarcinoma of the pancreas (CT TG01-01): a single-arm, phase 1/2 trial

**DOI:** 10.1038/s41416-020-0752-7

**Published:** 2020-02-17

**Authors:** Daniel H. Palmer, Juan W. Valle, Yuk Ting Ma, Olusola Faluyi, John P. Neoptolemos, Trine Jensen Gjertsen, Berit Iversen, Jon Amund Eriksen, Anne-Sophie Møller, Anne-Kirsti Aksnes, Robert Miller, Svein Dueland

**Affiliations:** 1 Liverpool Experimental Cancer Medicine Centre, Liverpool, UK; 2 The Clatterbridge Cancer Centre, Bebington, Wirral UK; 3 Division of Cancer Sciences, University of Manchester, Manchester, UK; 4 The Christie NHS Foundation Trust, Manchester, UK; 5 Queen Elizabeth Hospital, Birmingham, UK; 6 Institute of Immunology and Immunotherapy, University of Birmingham, Birmingham, UK; 7 Targovax ASA, Oslo, Norway; 8 The Norwegian Radium Hospital and Oslo University Hospital, Oslo, Norway

**Keywords:** Pancreatic cancer, Pancreatic cancer

## Abstract

**Background:**

TG01 is the first cancer immunotherapy targeting KRAS oncogenic mutations. This study assessed the safety and efficacy of TG01/GM-CSF in patients with resected pancreatic adenocarcinoma.

**Methods:**

Patients with stage I or II pancreatic adenocarcinoma who had undergone surgical resection (R0 or R1) received adjuvant gemcitabine with TG01/GM-CSF using two schedules of vaccination. Immune response was defined as a positive delayed-type hypersensitivity (DTH) response and/or positive T-cell proliferation assay.

**Results:**

Thirty-two patients were enrolled between February 2013 and May 2016. Nineteen were treated with the high antigen burden, with four serious adverse reactions considered possibly related to TG01 treatment, including three allergic reactions. On this basis, a further 13 patients received a modified vaccination schedule with reduced antigen burden, with no serious adverse events related to TG01. Ninety-five percent patients in the main cohort and 92% in the modified cohort had a positive immune response. Median overall survival (OS) was 33.1 months, and median disease-free survival (DFS) was 13.9 months for the main cohort. For the modified cohort, the median OS was 34.3 months and median DFS was 19.5 months.

**Conclusions:**

TG01/GM-CSF with gemcitabine was well tolerated, with high levels of immune activation. OS and DFS compare favourably with published data for adjuvant gemcitabine.

**Clinical trial registration:**

This clinical trial was registered at ClinicalTrials.gov (NCT02261714).

## Background

Pancreatic cancer is a major cause of cancer mortality globally, with a 5-year survival rate <5%. The 5-year survival rate improves to ~10% in patients who undergo surgical resection, and to ~20% with the addition of adjuvant chemotherapy. Based on the results of the ESPAC-3 and CONKO-001 trials,^1–3^ gemcitabine was established as adjuvant therapy, with 5-year survival of ~18–20%. In addition, it has been reported that there is no significant OS difference between patients treated with gemcitabine and 5-FU.^4^ More recently, two clinical trials have demonstrated a benefit from combination chemotherapy. Firstly, the ESPAC-4 study showed that the combination of gemcitabine and capecitabine improved survival compared with gemcitabine alone (hazard ratio (HR) 0.82 (95% CI, 0.68–0.98), *p* = 0.032) with 5-year survival rates of 28.8% vs 16.3% (*p* = 0.32),^5^ and this regimen is now included in the recently implemented ASCO clinical guidelines.^6^ Secondly, the PRODIGE 24/CCTG PA.6 study^7^ showed that adjuvant-modified (m)FOLFIRINOX was safe, and significantly improved disease-free survival (DFS) and overall survival (OS) when compared with gemcitabine. Median DFS was 12.8 months in the gemcitabine arm vs 21.6 months in the mFOLFIRINOX arm (HR = 0.59 (95% CI, 0.47–0.74)), and median OS was 34.8 vs 54.4 months (HR = 0.66 (95% CI, 0.49–0.89)), respectively, such that mFOLFIRINOX is now also considered a standard of care for patients sufficiently fit to tolerate this combination. Despite these recent advances, the majority of resected patients still ultimately develop disease recurrence, and novel therapies are still required.

The RAS family of human proto-oncogenes encode 21-kD guanine nucleotide-binding proteins (p21 RAS), which are key mediators involved in the regulation of cell growth and differentiation. The occurrence of oncogenic mutations in RAS in pancreatic cancer is very high.^8^ Moreover, expression of mutated RAS in different forms of cancer is associated with an overall worse prognosis and unresponsiveness to treatment.^9^ The fact that RAS mutations are expressed in a very high frequency of pancreatic adenocarcinomas (up to 90%) may be one of the reasons why standard chemotherapy and new ‘targeted' drugs tested against this form of cancer have mostly failed to significantly increase survival.

TG01 (Targovax ASA, Oslo, Norway) is the first injectable antigen-specific cancer immunotherapy (ASCI) targeted to treat patients with Kirsten rat sarcoma viral oncogene homolog (KRAS) mutations.^10^ TG01 consists of a mixture of 7 synthetic RAS peptides representing the 7 most common codon 12 and 13 oncogenic mutations in KRAS.

TG01 induces RAS-mutant-specific T-cell responses, which are enhanced by co-administration of GM-CSF (recombinant human granulocyte macrophage-colony-stimulating factor (Molgramostim)). The TG01 peptides are 17 amino acids long, and designed to activate both major histocompatibility complex MHC class II-restricted CD4 + helper T cells, as well as MHC class I-restricted CD8 + cytotoxic T cells, which are necessary to sustain the CD8 + cytotoxic T-cell effect.^10–12^ The activated CD4 + cells are also important for enhancing/facilitating cross-presentation of tumour neoantigens and tumour-associated antigens by dendritic cells (DC) at the site of the tumour, and thus broadening the cytotoxic CD8 + anticancer activity.

Several studies using one to seven RAS peptides have been performed previously, without adjuvant chemotherapy (trial design predating its routine use). Two of the studies using one or seven of the RAS peptides in patients who had undergone pancreatic resection were reported in a paper published by Wedén et al.^11^ The patients were administered the peptides on weeks 1, 2, 3, 4, 6 and 10 with boosters weekly for 4 weeks after 3 months or at 3 or 4 months for up to 2 years.

Serious drug reactions were not observed. In the 4-peptide study, 20/34 (58%) of patients mounted an immune response, whereas in the 7-peptide study (these are the same peptides used for the TG01-01 study) 12/12 (100%) of patients mounted an immune response. There were five patients who survived > 5 years, all of whom had responded to the RAS peptide immunisation.

As a result of these findings, it was concluded that the use of the seven peptides in future studies would account for the vast majority of mutations in pancreatic cancer, and that the induction and booster regimens suggested from these studies would be the basis of the initial vaccination regimen for the TG01-01 study.

At the time that this was planned, the standard-of-care chemotherapy used in the adjuvant setting was gemcitabine, and because it was not known if the use of this agent would interfere with the patients being able to mount and maintain an adequate immune response, and because it would not be ethical to deny patients an adjuvant chemotherapy, the study was planned to incorporate gemcitabine for up to six cycles starting within 12 weeks of surgery.

The initial vaccination regimen is based on earlier reported regimens used for peptide vaccines in clinical trials of pancreatic cancer.^10,11,13,14^ TG01 has earlier only been used as monotherapy, and the regimen was adjusted to include a phase of concurrent combination with the established adjuvant gemcitabine chemotherapy for resected pancreatic cancer.

It has been reported that several weekly peptide injections are required for mounting a detectable immune response, which is generally not observed until after at least 2–3 weeks of vaccination.^10^ For some patients, at least 6 weeks of vaccination is necessary for mounting a detectable immune response.^14^

After adjuvant treatment with gemcitabine, the median disease-free survival of pancreatic cancer patients that have undergone R0 and R1 resection is 14.3–15.2 months measured from surgery.^4,5^ It was therefore expected that the majority of the patients of this study would still be disease-free after completing the adjuvant gemcitabine treatment (8–9 months after surgery).

This study evaluated the safety, immunological response and clinical efficacy of TG01/GM-CSF with adjuvant gemcitabine chemotherapy, which was the standard of care at the time the study was conducted.

## Methods

### Patients

Patients at least 18 years of age with a confirmed diagnosis of stage I or II adenocarcinoma of the pancreas (according to AJCC staging) who had undergone successful surgical resection (R0 or R1), and were expected to receive gemcitabine as adjuvant chemotherapy within 12 weeks of surgery, were eligible for the study. Patients had to have acceptable laboratory test results, an Eastern Cooperative Oncology Group (ECOG) performance status (PS) of 0 or 1 and a life expectancy of at least 6 months.

Patients who had received prior therapy for pancreatic cancer, including radiation and chemotherapy (except for the primary resection or primary neoadjuvant chemotherapy), other investigational drugs within 4 weeks or any agent with a known effect on the immune system were excluded. Patients with any other malignancies within the previous 3 years (except for adequately treated carcinoma of the cervix or basal or squamous cell skin carcinoma) were also excluded.

The study was conducted at four centres in Norway and the United Kingdom (UK): Oslo University Hospital, the Clatterbridge Cancer Centre NHS Foundation Trust, the Christie NHS Foundation Trust and the Queen Elizabeth Hospital, Birmingham.

### Study design

This was a multicentre, phase I/II open-label study to assess the safety of TG01/GM-CSF vaccination in combination with adjuvant chemotherapy, and to assess the immune response and clinical efficacy of TG01/GM-CSF at 2 years in patients with resected pancreatic adenocarcinoma.

All patients included in the study were scheduled to receive adjuvant treatment with gemcitabine (note: patients could receive 5-fluorouracil (5-FU) instead of gemcitabine based on laboratory test results and usual practice at the study centre), and preferably to start TG01/GM-CSF vaccination at least 3 weeks before the start of the chemotherapy. Dexamethasone was used as an antiemetic with chemotherapy. The initial schedule (main cohort) of vaccination comprised TG01 (0.7 mg of intradermal injection (id)) together with GM-CSF (0.03 mg id) commencing as soon as possible after surgery, and given on days 1, 3, 5, 8, 15 and 22, and twice weekly thereafter until the completion of gemcitabine (starting within 12 weeks of surgery and given as 1000 mg/m^2^ on days 1, 8 and 15 of a 28-day cycle × 6 cycles). Thereafter, TG01/GM-CSF was given four weekly up to 1 year, and 12 weekly for up to 2 years. Four patients in the main cohort started vaccination at the same time as chemotherapy (i.e. received concomitant treatment as permitted by the protocol), and received TG01/GM-CSF on days 1, 3, 5, 8 and 15, and twice weekly thereafter until the completion of gemcitabine treatment. After 19 patients were treated, a modified TG01/GM-CSF dosing regimen was introduced. This was to investigate whether a reduced number of vaccinations, a reduced number of DTH tests used to detect immune responses and no vaccinations during chemotherapy treatment, could still induce the same immune responses while improving the safety profile, particularly with regard to allergic reactions seen in the main cohort. Patients in the modified cohort also received TG01/GM-CSF as soon as possible after surgery, administered on days 1, 8, 15, 22 and 36. Vaccination was then suspended until completion of gemcitabine treatment (six cycles). Thereafter, TG01 vaccination was given at weeks 4 and 5 post chemotherapy, and then followed the same schedule as for the main cohort.

### Outcomes

The primary outcome measures were safety and immune response. Safety was assessed by reported adverse events during the entire 2-year study period. Immune response to TG01 was assessed by two different antigen-specific assays: (1) delayed-type hypersensitivity (DTH test), and (2) in vitro T-cell proliferation.

The DTH test (measured up to nine times per patient) is a test in the skin measuring the presence of activated T cells recognising TG01. TG01 was injected intradermally, and the DTH test considered positive if the area of the skin reaction (redness/induration) at the injection site 48 h after injection had an average diameter ≥ 5 mm.

The T-cell proliferation assay is an in vitro assay showing proliferation response of TG01-specific T cells. Blood sampling and peripheral blood mononuclear cell (PBMC) isolation were performed on day 1 (baseline), week 11, week 52 and at the end of the study for the main cohort, and on day 1, week 8, 4 weeks after the last chemotherapy injection, week 52 and at the end of the study for the modified cohort. T-cell responses were considered positive if the stimulation index (SI) was ≥ 2, indicating an increase in proliferation of TG01-specific T cells after stimulation with TG01 compared with unstimulated cells.

An immune responder was defined as having either a positive DTH response and/or positive T-cell proliferation.

Secondary outcome measures were DFS and OS at 1 and 2 years measured from the date of surgery. Patients still disease-free at the last computed tomography (CT) scan collected in the study were censored on the date of the last CT scan. Patients still alive when last contacted in the study were censored at the date of the last contact.

Exploratory endpoints included assessment of the relationship between KRAS status (in resected primary tumour) and survival outcomes, and changes in CA19-9 levels.

### Statistical analysis

Safety and survival endpoints were analysed by using the All Treated Patients (ATP) analysis set (defined as patients who received at least one dose of IMP (TG01, GM-CSF, gemcitabine or 5-FU/leucovorin)). All enrolled patients did receive at least one dose of IMP. Immune response endpoints were analysed using the Immune Analysis Set (IAS, defined as patients who provided at least one set of T-cell and/or DTH response results).

Summary statistics for continuous variables were presented by the number of patients (*n*), mean, standard deviation (SD), median, interquartile range, minimum and maximum. Categorical variables included frequency counts, and the number and percentage of patients (*n* (%)). In general, the denominator for percentage calculations is the number of patients in the analysis set. However, for tables by time point the percentages were calculated by using the number of patients with available data at that time point.

DFS and OS were estimated using the Kaplan–Meier method, and are also presented by swimmer plots.

## Results

### Patients

A total of 32 patients were enrolled between February 2013 and May 2016, and followed until the last patient included had been in the study for 3 years. Baseline characteristics are summarised in Table 1. Nineteen patients were included in the main cohort (including 4 patients who received concomitant treatment), and 13 patients were included in the modified cohort.

**Table 1:** Baseline characteristics.

Parameter	Main cohort	Modified cohort	Overall
	(*n* = 19)	(*n* = 13)	(*N* = 32)
*Age (years)*			
Median (min, max)	67 (49, 79)	59 (46, 74)	65 (46, 79)
*Gender,* n *(%)*			
Male	10 (53%)	11 (85%)	21 (66%)
Female	9 (47%)	2 (15%)	11 (34%)
*ECOG,* n *(%)*			
0	8 (42%)	6 (46%)	14 (44%)
1	11 (58%)	7 (54%)	18 (56%)
*CA19-9 (U/ml)*			
Median (min, max)	15 (5, 240)	25 (9, 2166)	16 (5, 2166)
*Haemoglobin (g/L)*			
Median (min, max)	124 (104, 153)	127 (109, 148)	124.5 (104, 153)
*Disease staging at diagnosis*			
T stage			
T1	1 (5%)	0	1 (3%)
T2	1 (5%)	0	1 (3%)
T3	17 (90%)	13 (100%)	30 (94%)
N stage			
N0	7 (37%)	2 (15%)	9 (28%)
N1	12 (63%)	11 (85%)	23 (72%)
M stage			
M0	19 (100%)	13 (100%)	32 (100%)
*Resection surgical outcome,* n *(%)*			
R0	6 (32%)	4 (31%)	10 (31%)
R1	13 (68%)	9 (69%)	22 (69%)
*KRAS mutation detected,* n *(%)*			
Yes	16 (84%)	10 (77%)	26 (81%)
No	3 (16%)	3 (23%)	6 (19%)
*Time from surgery to the first IMP administration (weeks)*			
Median (min, max)	8 (7, 12)	9 (7, 12)	9 (7, 12)

A total of 28 patients discontinued study treatment due to the following reasons: adverse events (five patients), death (two patients due to pneumonia and disease progression, not treatment related), consent withdrawn (four patients) and investigator decision (two patients). Fifteen patients discontinued study treatment due to disease recurrence as stipulated in the protocol.

Thirty one of the 32 patients reported concomitant medical conditions in addition to pancreatic cancer when they were included in the study. Overall, the most frequently reported concomitant medical conditions were hypertension, type 2 diabetes mellitus, constipation, hypercholesterolaemia, gastro-oesophageal reflux disease and fatigue.

Pathological assessment of the resected specimens showed that 69% of patients had R1, and 31% had R0 pancreatic resections.

The initial polymerase chain reaction (PCR) test intended for the identification of KRAS mutation was performed on biopsy prior to study enrolment, and showed that 6/32 (19%) patients did not have a KRAS mutation. However, cancer-related mutant KRAS cell-free DNA analysis revealed that only 2/32 (6%) did not have KRAS mutation detected. In conclusion, these results indicate that 30 out of the 32 patients had KRAS mutation.

### Treatment

The overall median number of TG01/GM-CSF injections was 14. Since the number of planned vaccinations was higher for the main cohort, the median number of injections for this cohort was 18 compared with 12 for the modified cohort. Patients also received TG01 (without GM-CSF) for DTH purposes, and the median numbers of injections for DTH were seven and three for the main and modified cohort, respectively. The planned number of DTH tests for the two cohorts were nine and three, respectively.

The median number of gemcitabine cycles received overall was six. The median number of cycles for the main and modified cohorts were four and six, respectively. Two patients in the main cohort initially received gemcitabine, and subsequently received 5-FU. The reason for changing to 5-FU was for one patient elevated alanine aminotransferase and the other a SAE of anaphylactic shock related to concomitant medication Aprepitant (Emend^®^).

### Safety

Twenty treatment-emergent serious adverse events (TESAEs) were reported in 11/32 patients. Of these, ten serious adverse reactions (SARs) were reported in 7/32 patients; six reactions were related to gemcitabine (anaemia, pulmonary infection, pulmonary embolism, transient ischaemic attack and two fever), three related to TG01 treatment (two anaphylaxes and one hypersensitivity) and one possibly related to all products (dyspnoea). All the three allergic reactions related to TG01 treatment occurred after several cycles of gemcitabine, exclusively in the main cohort, and resolved within 1–2 h, requiring oral steroids and antihistamines only. Prophylactic safety measures were implemented in the main cohort for the ongoing patients after the allergic reactions were reported. Thereafter, the modified cohort was introduced in the study, and prophylaxis in this cohort was not required. In the modified cohort, there were only two serious adverse reactions (pulmonary embolism and transient ischaemic attack), both considered related to chemotherapy, and no serious allergic reactions or SAEs related to TG01 immunotherapy were reported. In the modified vaccination group, although prophylaxis was available if the patient experienced an event of hypersensitivity reaction, this was not required at any stage of the study. There were no treatment-related deaths in this study.

Of the 528 reported treatment-emergent adverse events (TEAEs), 90 events in 23 patients were reported to be related to TG01 immunotherapy. The majority of the TEAEs were related to Grade 1 and 2 General Disorders and Administration Site Reactions such as erythema, pruritus, oedema, pain, fatigue and flu-like symptoms. All related events were Grade 1 or 2, except for the two reported anaphylactic reactions that were Grade 4. In total, 61 TEAEs Grade 3 or 4 were reported (Table 2). Gemcitabine is known to produce skin rash and flu-like symptoms (very common, ≥ 1/10 according to the summary of product characteristics), and in the study one event (grade 2) of rash and four events (grade 1) of influenza-like illness were reported as related to gemcitabine only.

**Table 2:** Treatment-emergent adverse events Grade 3 and 4 by SOC and preferred term.

SOC	Grade 3		Grade 4	
Adverse event	Patients	Events	Patients	Events
Any adverse event	23	55	5	6
*Blood and lymphatic system disorders*				
Neutropenia	10	11	1	1
Anaemia	1	1		
Thrombocytopenia	1	1		
*Gastrointestinal disorders*				
Abdominal pain	3	3		
Diarrhoea	2	2		
Abdominal pain upper	1	1		
*General disorders and administration site conditions*				
Fatigue	2	2		
*Immune system disorders*				
Anaphylactic reaction			2	2
Anaphylactic shock	1	1		
*Infections and infestations*				
Biliary sepsis	1	1		
Upper respiratory tract infection	1	1		
Urosepsis	1	1		
*Injury, poisoning and procedural complications*				
Alcohol poisoning	1	1		
Femoral neck fracture	1	1		
*Investigations*				
Neutrophil count decreased	6	9	1	1
Haemoglobin decreased	1	1		
Platelet count decreased	1	1		
*Metabolism and nutrition disorders*				
Hyperglycaemia	1	1	2	2
Diabetes mellitus	1	1		
Hyperkalaemia	1	1		
Hypokalaemia	1	1		
Hyponatraemia	1	1		
*Nervous system disorders*				
Coordination abnormal	1	1		
*Psychiatric disorders*				
Depression	1	1		
*Respiratory, thoracic and mediastinal disorders*				
Pulmonary embolism	2	2		
*Vascular disorders*				
Hypertension	7	8		

### Immunological response

Immune responses, as detected by a positive DTH test and T-cell proliferation (SI ≥ 2), are summarised in Table 3. In total, 30/32 (94%) patients had a positive immune response. A total of 18/19 (95%) and 12/13 (92%) patients had a positive immune response in the main and modified cohorts, respectively.

**Table 3:** Immune response during the entire study period.

Parameters	Main cohort (*n* = 19)	Modified cohort (*n* = 13)	Overall (*N* = 32)
Immune responders	18 (95%)	12 (92%)	30 (94%)
DTH positive	18 (95%)	8 (62%)	26 (81%)
mutRAS-specific T cells	14 (74%)	12 (92%)	26 (81%)

The high rate of positive immune responses demonstrates that TG01/GM-CSF vaccination effectively induces TG01-specific T cells in peripheral blood.

As only two patients were not immune responders, no correlation with clinical efficacy is possible.

### Efficacy

**Fig. 1:**
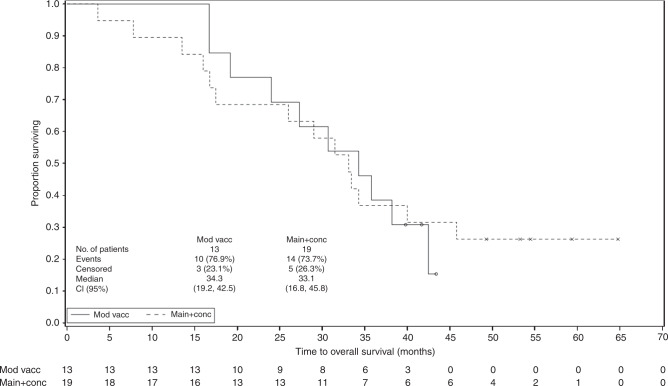
**OS by treatment cohort**.

Survival data are summarised in Table 4 and Fig. 1.

**Table 4:** Survival rates at 1, 2 and 3 years (assessed from surgery).

	Survival rate at 1 year			Survival rate at 2 years			Survival rate at 3 years		
	Main cohort (*n* = 19)	Modified cohort (*n* = 13)	Overall (*N* = 32)	Main cohort (*n* = 19)	Modified cohort (*n* = 13)	Overall (*N* = 32)	Main cohort (*n* = 19)	Modified cohort (*n* = 13)	Overall (*N* = 32)
*n* (%)	17 (89.5%)	13 (100.0%)	30 (93.8%)	13 (68.4%)	10 (76.9%)	23 (71.9%)	7 (36.8%)	5 (38.5%)	12 (37.5%)
95% CI	(75.7, 100.0)	(100.0, 100.0)	(85.4, 100.0)	(47.5–89.3)	(54.0–99.8)	(56.3, 87.5)	(15.2, 58.5)	(12.0, 64.9)	(20.7, 54.3)

For the main cohort, median OS was 33.1 months (95% confidence interval (CI) 16.8, 45.8), and median DFS was 13.9 months (95% CI 4.5, 21.0). For the modified cohort, median OS was 34.3 months (95% CI 19.2, 42.5), while median DFS was 19.5 months (95% CI 9.7, not calculable). For the total study population, median OS was 33.3 months (95% Cl 24.0, 40.0) and median DFS was 16.1 months (95% CI 11.1–19.6).

A subgroup analysis was performed on the association between completed chemotherapy and OS. For patients who received five or six cycles of chemotherapy, the median OS was 45.8, 34.3 and 37.0 months, in the main (*n* = 11 patients), modified (*n* = 11 patients) and total (*n* = 22 patients) study population, respectively.

**Fig. 2:**
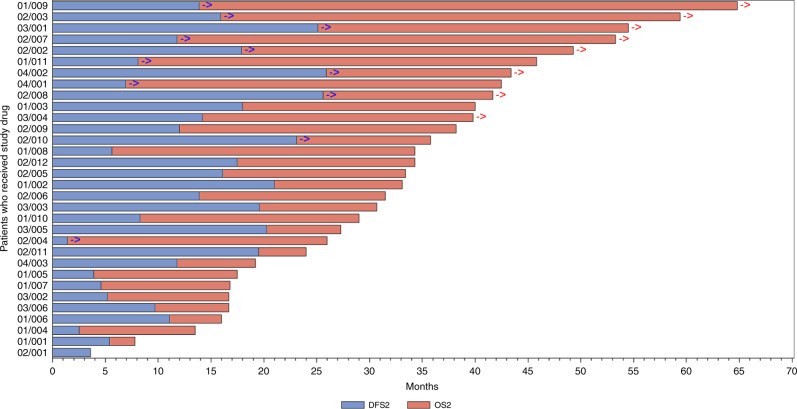
OS was assessed until the last patient had been in the study for 3 years. The blue arrow indicates that the patient had been censored for DFS, meaning the patient was disease-free at the last CT scan collected in the study. The red arrow indicates that the patient had been censored for OS, meaning the patient was alive at the last contact collected in the study.

A swimmer’s plot of DFS and OS is provided in Fig. 2.

## Discussion

This study has demonstrated that TG01/GM-CSF vaccination, when used in conjunction with adjuvant chemotherapy, is generally well tolerated. The only significant safety finding related to TG01 was hypersensitivity in some patients, which appeared to be induced after the patients have received several cycles of chemotherapy while concurrently receiving TG01 vaccinations. Five patients (26.3%) in the main cohort experienced TEAEs associated with allergic reactions; no patients in the modified cohort, with a reduced antigen burden, and a break from vaccination during chemotherapy, experienced these types of TEAEs indicating a more acceptable level of toxicity. It is possible that the total peptide load of vaccinations plus DTH tests may have contributed to the risk of hypersensitivity in the main cohort, and it appears that the mandatory prophylaxis introduced for patients in this cohort reduced the risk of further severe episodes. A cohort with a modified vaccination schedule and fewer DTH assessments was introduced, and this appears to have largely abrogated the risk of hypersensitivity reactions without the need for prophylaxis, with no significant reactions reported in patients receiving this schedule. Some low-grade local reactions, however, were inevitably observed, and likely a sign of immune activation.

The incidence of Grade 4 TEAEs was low in general, with five patients (26.3%) in the main cohort and no patients in the modified cohort experiencing these. One patient (main cohort) died due to a TEAE, pneumonia, which was not considered related to the investigational medicinal product (IMP). The results of laboratory assessments did not indicate a clinically significant difference between the dosing regimens, and the majority of TEAEs associated with laboratory assessments were considered related to chemotherapy only.

Ninety-four percent of the patients had at least one reported immune response during the study period. With the exception of one patient in the main cohort and one patient in the modified cohort, all patients had at least one reported immune response. This high level of immune activation is comparable with immune activation observed in studies with similar mutant-RAS peptide vaccination monotherapy,^11^ which demonstrates that TG01 vaccination can successfully be used in combination with chemotherapy.

With 3 years of follow-up, the emerging durability data on OS and DFS in this trial are encouraging when viewed in the context of current literature reporting large-scale trials with gemcitabine as monotherapy, which range from 10.7 to 13 months for DFS, and 17.1 to 26.5 months for median survival.^4,5,15–19^

Based on time from surgery, in this study median DFS was 16.1 months and median OS was 33.3 months for the total study population, which compare favourably with published data for gemcitabine monotherapy, and indeed even with gemcitabine and capecitabine combination therapy (GemCap). Modification of the vaccination schedule, whilst appearing to eliminate the risk of allergic reactions, did not appear to compromise this encouraging efficacy.

A previous study suggested an association between the amount of chemotherapy and OS.^20^ In this study, median OS was 28.0 months (CI 26.1, 30.9) for those who completed six cycles of gemcitabine, or 5-FU and 14.6 months (CI 12.5, 16.9) for those who received less than six cycles. Our subgroup analysis showed that a similar trend with median OS for patients receiving five or six cycles of gemcitabine (or 5-FU) in combination with TG01 was 37.0 months in this subpopulation (*n* = 22). These findings may suggest synergism between TG01/GM-CSF and chemotherapy.

In conclusion, this pilot study demonstrated that TG01/GM-CSF vaccination produces an appropriate immune response, and is safe to use in conjunction with adjuvant chemotherapy. The study has also identified the vaccination schedule for future development of TG01 in pancreatic cancer in combination with chemotherapy. The clinical efficacy endpoints of DFS and OS compare favourably to historical data reported. Since this study was conducted, recent clinical trials have demonstrated improved survival with the use of combination chemotherapy (GemCap or mFOLFIRINOX) in the adjuvant setting, in patients sufficiently fit to receive these in the post-operative setting. Nevertheless, the majority of patients still experience disease recurrence, and further therapeutic advances are required. Thus, the incorporation of a safe, well-tolerated, non-cytotoxic agent such as TG01 may be particularly attractive in this context, and based on the encouraging safety, immunological and efficacy data presented here.

## Data Availability

The data in this publication are commercially confidential. Data requests should be made to the corresponding author.
